# Predictive Biomarkers of Immune Checkpoint Inhibitor-Based Mono- and Combination Therapies for Hepatocellular Carcinoma

**DOI:** 10.7150/jca.90128

**Published:** 2024-01-01

**Authors:** Long-Bin Jeng, John Wang, Chiao-Fang Teng

**Affiliations:** 1 Organ Transplantation Center, China Medical University Hospital, Taichung, Taiwan.; 2 Department of Surgery, China Medical University Hospital, Taichung, Taiwan.; 3 Cell Therapy Center, China Medical University Hospital, Taichung, Taiwan.; 4 Department of Pathology, China Medical University Hospital, Taichung, Taiwan.; 5 Graduate Institute of Biomedical Sciences, China Medical University, Taichung, Taiwan.; 6 Program for Cancer Biology and Drug Development, China Medical University, Taichung, Taiwan.; 7 Research Center for Cancer Biology, China Medical University, Taichung, Taiwan.

**Keywords:** hepatocellular carcinoma, immune checkpoint inhibitor, mono-therapy, combination therapy, predictive biomarker

## Abstract

Hepatocellular carcinoma (HCC) is among the most frequent and deadly human cancers worldwide. It has been shown that interaction between immune checkpoint receptors and ligands plays a crucial role in inhibition of T cell-mediated anti-tumor immune responses, thereby assisting tumor cells to evade the host immune surveillance. Therefore, several immune checkpoint inhibitors (ICIs) that selectively block immune checkpoint receptors or ligands have been developed as clinically effective and safe immunotherapeutic agents for treating HCC, including the inhibitors targeting cytotoxic T lymphocyte-associated antigen 4, programmed death 1, and programmed death ligand 1. In addition, various combinations of ICIs and other ICIs or tyrosine kinase inhibitors or vascular endothelial growth factor inhibitors have also emerged as clinically beneficial treatments for HCC. However, the overall response rates of ICI mono-therapy and combination therapy in HCC patients remain unsatisfied, highlighting the urgent need for discovering valuable predictive biomarkers to achieve personalized therapy. This review comprehensively summarizes the literature-based evidence validating a variety of biomarkers with predictive significance for treatment responses and outcomes in HCC patients receiving various ICI-based mono- and combination therapies.

## Introduction

As the predominant type of primary liver cancer, hepatocellular carcinoma (HCC) accounts for over 90% of primary liver malignancies and is the sixth most prevalent and the third most deadly human cancer worldwide, contributing to approximately 900,000 cases and 800,000 deaths per year 1-3. Many therapeutic options have been well established for treating HCC, including surgical therapies (such as liver transplantation and resection) 4, 5, locoregional therapies (such as radiotherapy, ablation, and embolization) 6, 7, and systemic therapies (such as chemotherapy and molecular targeted therapy) 8, 9. Moreover, immunotherapies such as immune checkpoint inhibitor (ICI) therapy have been developed as a promising treatment modality for HCC 10, 11. However, the therapeutic efficacy of these treatment modalities varies among patients and remains to be improved. Therefore, the discovery of valuable biomarkers for predicting therapeutic responses and outcomes in HCC patients is an important goal to select the most suitable patient for the most suitable treatment (the so-called personalized therapy) to improve patient survival.

Immune checkpoint molecules include the co-inhibitory receptors expressed by effector T cells and the corresponding ligands expressed by tumor cells and stromal cells 12, 13. Through the interaction between the receptors and ligands, immune checkpoint molecules play an important role in suppression of the activation and function of effector T cells, thereby facilitating tumor cell escape from T cell-mediated anti-tumor immune responses 14, 15. As a result, many monoclonal antibodies that block the binding of immune checkpoint receptors to ligands (the so-called ICIs) have been generated as effective immunotherapeutic agents to restore T cell-mediated killing of tumor cells 16, 17. The ICIs that have been licensed or are in clinical research for HCC treatment include the agents targeting the co-inhibitory receptors such as cytotoxic T lymphocyte-associated antigen 4 (CTLA-4) and programmed death 1 (PD-1) and the agents targeting the ligand of PD-1, programmed death ligand 1 (PD-L1) 18-20. In addition, the combination of ICIs targeting CTLA-4 and PD-1 or PD-L1, the combination of ICIs targeting PD-1 or PD-L1 and tyrosine kinase inhibitors (TKIs), and the combination of ICIs targeting PD-L1 and vascular endothelial growth factor (VEGF) inhibitors have been also licensed or are under clinical validation for HCC therapy 18-20. It has been shown that ICI-based mono- and combination therapies are active, tolerate, and clinically beneficial against HCC, although patients may concurrently receive various locoregional therapies. However, the overall response rates remain unsatisfied in HCC patients, with only about 15% and 30% for ICI-based mono- and combination therapies, respectively 18-20. Therefore, the development of clinically useful predictive biomarkers for identifying HCC patients who are more likely to respond to ICIs is urgently needed to advance personalized therapy for better patient outcomes.

This review provides a comprehensive summary of the hitherto published literature, which unravel various promising biomarkers at pre-treatment, on-treatment, and post-treatment time points in tissue, blood, and stool samples for predicting the therapeutic responses and clinical benefits of different categories of ICI-based therapies in HCC patients, including ICI mono-therapy and combination therapy with other ICIs or TKIs or VEGF inhibitors.

## Predictive biomarkers of ICI mono-therapy for HCC

Several biomarkers have been validated with predictive value in HCC patients receiving ICI mono-therapy in multiple lines of studies (Table 1). The study conducted by Sangro et al. 21 analyzed the pre-treatment expression of PD-L1 in tumor tissues of 195 HCC patients treated with PD-1 ICI (nivolumab) and showed that high PD-L1 expression level (≥ 1%) was associated with better median overall survival (OS) (28.1 versus (vs.) 16.6 months, P value = 0.03) than low PD-L1 expression level (< 1%). The study conducted by Feun et al. 22 evaluated the pre-treatment levels of a panel of cytokines and chemokines in blood of 28 HCC patients treated with PD-1 ICI (pembrolizumab) and identified that low transforming growth factor-beta (TGF-β) level (< 200 pg/mL) predicted longer median OS (not reached (NR) vs. 7 months, P value = 0.005) and progression-free survival (PFS) (NR vs. 2 months, P value = 0.008) than high TGF-β level (≥ 200 pg/mL). The study conducted by Zhang et al. 23 combined the pre-treatment blood levels of C-reactive protein (CRP) and alpha-fetoprotein (AFP) to stratify 101 HCC patients treated with PD-1 ICI (such as nivolumab, toripalimab, sintilimab, and pembrolizumab) into low-risk (CRP ≤ 20.9 mg/L and AFP ≤ 400 ng/mL), medium-risk (CRP > 20.9 mg/L or AFP > 400 ng/mL), and high-risk (CRP > 20.9 mg/L and AFP > 400 ng/mL) subgroups and confirmed that patients with a low-risk score had the highest disease control rate (DCR) (82% vs. 65% vs. 35%, P value = 0.002) and the best OS (P value < 0.001) and PFS (P value < 0.001) compared with patients with a medium- or high-risk score. The study conducted by Dong et al. 24 combined the pre-treatment albumin-bilirubin (ALBI) grade and age to stratify 38 HCC patients treated with PD-1 ICI (such as sintilimab and camrelizumab) combined with locoregional therapy (such as conventional radiotherapy (CRT), hypofractionated radiotherapy (HFRT), and stereotactic body radiotherapy (SBRT)) into low-risk (ALBI grade 1 and age ≥ 53 years) and high-risk (ALBI grade 2 and age < 53 years) subgroups and revealed that patients with a low-risk score had higher objective response rate (ORR) (50% vs. 14%, P value = 0.001) and longer median OS (NR vs. 10.1 months, P value = 0.003) and PFS (15.3 vs. 2.7 months, P value < 0.001) than patients with a high-risk score. In addition, the study conducted by Choi et al. 25 measured the pre-treatment counts of neutrophils and lymphocytes in blood of 194 HCC patients treated with PD-1 ICI (nivolumab) and indicated that low neutrophil to lymphocyte ratio (NLR) (< 3) was correlated with longer median OS (61.3 vs. 21.0 weeks, P value < 0.001) and PFS (11.0 vs. 7.1 weeks, P value = 0.01) than high NLR (≥ 3). The study conducted by Hung et al. 26 also calculated the NLR in blood of 45 HCC patients treated with PD-1 ICI (nivolumab) and verified that low pre**-**treatment NLR (≤ 2.5), on**-**treatment NLR (< 4.1), or post**-**treatment NLR (≤ 2.7) predicted DCR (sensitivity/specificity, 57%/97%; 86%/58%; 64%/87%, respectively) and longer PFS (P value = 0.004; P value = 0.006; P value = 0.001, respectively) than high pre**-**treatment NLR (> 2.5), on**-**treatment NLR (≥ 4.1), or post**-**treatment NLR (> 2.7). The study conducted by Dharmapuri et al. 27 detected both NLR and platelet to lymphocyte ratio (PLR) in blood of 103 HCC patients treated with PD-1 ICI (nivolumab) combined with or without locoregional therapy (such as transarterial chemoembolization (TACE) and transarterial radioembolization (TARE)) and showed that low pre-treatment or post-treatment NLR (all < 5) predicted longer median OS (23 vs. 10 months, P value = 0.004; 35 vs. 9 months, P value < 0.001, respectively) and PFS (16 vs. 5 months, P value = 0.022; 35 vs. 5 months, P value < 0.001, respectively) than high NLR (≥ 5). When PLR was divided into low (≤ 118 for pre-treatment; ≤ 125 for post-treatment), medium (> 118 to < 224 for pre-treatment; > 125 to < 229 for post-treatment), and high (≥ 224 for pre-treatment; ≥ 229 for post-treatment) level groups, patients with a low pre-treatment or post-treatment PLR had the longest median OS (35 vs. 10 vs. 15 months, P value = 0.05; NR vs. 19 vs. 10 months, P value = 0.013, respectively) compared with patients with a medium or high PLR. The study conducted by Huang et al. 28 determined the pre-treatment NLR, PLR, systemic immune-inflammation index (SII), and lymphocyte to monocyte ratio (LMR) in blood of 110 HBV-related HCC patients treated with PD-1 ICI and demonstrated that low NLR (< 5), PLR (< 140), or SII (< 970) or high LMR (≥ 1.8) was associated with longer median OS (7.3 vs. 6.0 months, P value = 0.0007; 7.3 vs. 6.7 months, P value = 0.0029; 7.2 vs. 5.9 months, P value < 0.0001; 7.2 vs. 6.7 months, P value = 0.0038, respectively) and PFS (6.7 vs. 5.4 months, P value = 0.0013; 6.7 vs. 5.9 months, P value = 0.0016; 6.7 vs. 4.5 months, P value < 0.0001; 6.8 vs. 5.5 months, P value = 0.0006, respectively) than high NLR (≥ 5), PLR (≥ 140), or SII (≥ 970) or low LMR (< 1.8). The study conducted by Jeon et al. 29 measured the frequency of classical monocytes (cMonocyte) and PD-L1-expressing classical monocytes (cMonocyte-PDL1) in blood of 45 HCC patients treated with PD-1 ICI (nivolumab) and calculated the on-treatment monocyte index by dividing cMonocyte_D7/D0_ by cMonocyte-PDL1_D7/D0_, in which cMonocyte_D7/D0_ and cMonocyte-PDL1_D7/D0_ were defined as the fold change in the frequency of cMonocyte and cMonocyte-PDL1 at day 7 over day 0 after treatment initiation, respectively. It was shown that high monocyte index (≥ 1) predicted DCR (sensitivity/specificity, 83%/65%) and better PFS (P value = 0.008) than low monocyte index (< 1). Moreover, the study conducted by Mao et al. 30 analyzed the pre-treatment composition of gut microbiota in stool of 30 HCC patients treated with PD-1 ICI and ascertained that patients with a high abundance of Erysipelotrichaceae bacterium-GAM79 or low abundance of Veillonellaceae had longer median OS (NR vs. 15.9 months, P value = 0.041; NR vs. 7.8 months, P value = 0.03, respectively) and PFS (15.9 vs. 5.5 months, P value = 0.021; 10.8 vs. 3.6 months, P value = 0.005, respectively) than patients with a low abundance of Erysipelotrichaceae bacterium-GAM79 or high abundance of Veillonellaceae.

## Predictive biomarkers of ICI combination therapy with other ICIs or TKIs for HCC

The predictive value of many biomarkers in HCC patients receiving ICI combination therapy with other ICIs or TKIs have been validated in multiple lines of studies (Table 2). The study conducted by Ng et al. 31 investigated the pre-treatment expression of CD38 in tumor tissues of 49 HCC patients treated with PD-1 or PD-L1 ICI combined with or without CTLA-4 ICI and showed that high CD38-positive (CD38^+^) cell proportion (≥ 5%) was associated with higher ORR (43% vs. 4%, P value = 0.019) and longer median OS (19.1 vs. 9.6 months, P value = 0.0295) and PFS (8.2 vs. 1.6 months, P value = 0.0065) than low CD38^+^ cell proportion (< 5%). The study conducted by Muhammed et al. 32 measured the pre-treatment prognostic nutritional index (PNI), NLR, and PLR in blood of 362 HCC patients treated with PD-1 ICI (such as nivolumab and pembrolizumab) or PD-L1 ICI (such as atezolizumab, avelumab, and durvalumab) combined with or without CTLA-4 ICI (ipilimumab) and revealed that high PNI (≥ 45) predicted higher DCR (66% vs. 52%, P value = 0.014) and longer median OS (17.7 vs. 10.8 months, P value = 0.018) than low PNI (< 45); low NLR (< 5) predicted higher ORR (22% vs. 12%, P value = 0.034) and longer median OS (17.6 vs. 7.7 months, P value = 0.0001) and PFS (3.8 vs. 2.1 months, P value = 0.025) than high NLR (≥ 5); low PLR (< 300) predicted longer median OS (16.5 vs. 6.4 months, P value < 0.0001) and PFS (3.7 vs. 1.8 months, P value = 0.0006) than high PLR (≥ 300). In addition, the study conducted by Shao et al. 33 detected the on-treatment change in AFP levels in blood of 43 HCC patients treated with PD-1 ICI combined with or without CTLA-4 ICI and identified that high AFP decrease (> 20%) predicted higher ORR (73% vs. 14%, P value < 0.001) and DCR (80% vs. 46%, P value = 0.033) and longer median OS (28.0 vs. 11.2 months, P value = 0.048) and PFS (15.2 vs. 2.7 months, P value = 0.002) than low AFP decrease (≤ 20%). The studies conducted by Lee et al. 34 and Hsu et al. 35 also evaluated the on-treatment AFP change in blood of patients. The former study confirmed that high AFP decrease (> 10%) predicted higher ORR (64% vs. 10%, P value < 0.001) and DCR (82% vs. 14%, P value < 0.001) and longer median OS (24.7 vs. 6.9 months, P value = 0.009) than low AFP decrease (≤ 10%) in 75 HCC patients treated with PD-1 ICI (such as nivolumab and pembrolizumab) combined with or without TKI (such as sorafenib, regorafenib, and lenvatinib); the latter study verified that high AFP decrease (> 15%) predicted higher ORR (46% vs. 10%, P value < 0.001) and DCR (80% vs. 29%, P value < 0.001) and longer median OS (21.9 vs. 5.6 months, P value < 0.001) and PFS (7.5 vs. 2.3 months, P value < 0.001) than low AFP decrease (≤ 15%) in 95 HCC patients treated with PD-1 ICI (such as nivolumab and pembrolizumab) combined with or without locoregional therapy (such as SBRT, TACE, and radiofrequency ablation (RFA)) or TKI (such as sorafenib, regorafenib, and lenvatinib). The study conducted by Sun et al. 36 analyzed the post-treatment change in AFP and protein induced by vitamin K absence or antagonist-II (PIVKA-II) levels in blood of 235 HCC patients treated with PD-1 ICI (such as nivolumab, toripalimab, sintilimab, camrelizumab, and pembrolizumab) combined with or without locoregional therapy (TACE) and/or TKI and demonstrated that high AFP decrease (> 50%) or PIVKA-II decrease (> 50%) was correlated with higher ORR (53% vs. 18%, P value < 0.001; 50% vs. 18%, P value = 0.003, respectively) and longer median OS (NR vs. 13.7 months, P value = 0.003; NR vs. 14.4 months, P value = 0.006, respectively) and PFS (13.1 vs. 4.5 months, P value < 0.001; 10.9 vs. 4.5 months, P value = 0.021, respectively) than low AFP decrease (≤ 50%) or PIVKA-II decrease (≤ 50%). The study conducted by Li et al. 37 established a nomogram based on 7 pre-treatment clinical parameters including Eastern Cooperative Oncology Group performance status (ECOG PS), TACE, extrahepatic metastasis (EHM), Child-Pugh score, alanine aminotransferase (ALT), AFP, and PLR to stratify 258 HCC patients treated with PD-1 ICI (such as sintilimab and camrelizumab) combined with or without TKI (such as sorafenib, regorafenib, and lenvatinib) and ascertained that patients with a low-risk score (≤ 182.7) had the longest median OS (53.2 vs. 17.5 vs. 7.6 months, P value < 0.0001) compared with patients with a medium-risk (> 182.7 to ≤ 240.3) or high-risk (> 240.3) score. The study conducted by Guo et al. 38 combined the pre-treatment PIVKA-II level and metastasis to stratify 191 HCC patients treated with PD-1 ICI (such as nivolumab, toripalimab, sintilimab, pembrolizumab, and tislelizumab) combined with TKI (lenvatinib) into low-risk (PIVKA-II < 600 mAU/mL and without metastasis), medium-risk (PIVKA-II > 600 mAU/mL or with metastasis), and high-risk (PIVKA-II > 600 mAU/mL and with metastasis) subgroups and found that patients with a low-risk score had the longest median OS (24.0 vs. 17.7 vs. 12.1 months, P value < 0.001) compared with patients with a medium- or high-risk score.

Moreover, the study conducted by Xu et al. 39 examined the pre-treatment levels of circulating tumor DNA (ctDNA) tumor mutation burden (TMB) and maximum somatic allele frequency (MSAF) in blood of 107 HCC patients treated with PD-1 ICI (camrelizumab) combined with TKI (apatinib). It was shown that low ctDNA TMB (≤ 4) predicted higher DCR (90% vs. 64%, P value = 0.002) and longer OS (P value = 0.019) than high ctDNA TMB (> 4); low ctDNA MSAF (≤ 0.027) predicted higher DCR (90% vs. 72%, P value = 0.043) and better OS (P value = 0.002) and PFS (P value = 0.004) than high ctDNA MSAF (> 0.027). The study conducted by Su et al. 40 determined the pre-treatment counts of PD-L1^+^ circulating tumor cells (CTCs) in blood of 47 HCC patients treated with PD-1 ICI (such as sintilimab, camrelizumab, and tislelizumab) combined with locoregional therapy (intensity-modulated radiotherapy (IMRT)) and TKI (such as sorafenib, regorafenib, lenvatinib, apatinib, and anlotinib) and unraveled that low PD-L1^+^ CTC count (< 2) predicted higher ORR (sensitivity/specificity, 77%/67%; 57% vs. 17%, P value = 0.007) and longer median OS (NR vs. 10.8 months, P value = 0.001) than high PD-L1^+^ CTC count (≥ 2). The study conducted by Lee et al. 41 monitored the pre-treatment composition of gut microbiota in stool of 74 HCC patients treated with PD-1 ICI (such as nivolumab and pembrolizumab) combined with or without TKI (41 patients as derivation cohort and 33 patients as validation cohort) and stratified patients into good-signature (coexistence of Prevotella 9 depletion and Lachnoclostridium enrichment), fair-signature (coexistence of both two bacteria depletion or both two bacteria enrichment), and poor-signature (coexistence of Prevotella 9 enrichment and Lachnoclostridium depletion) subgroups. In the derivation cohort, patients with a good-signature microbiota had the longest median OS (22.8 vs. 8.0 vs. 4.8 months, P value = 0.007) compared with patients with a fair- or poor-signature microbiota; in the validation cohort, patients with a good-signature microbiota had the highest ORR (53% vs. 20% vs. 0%, P value = 0.06) and DCR (95% vs. 90% vs. 0%, P value < 0.001) and the longest median OS (NR vs. 11.1 vs. 6.5 months, P value < 0.001) and PFS (8.8 vs. 7.6 vs. 1.8 months, P value < 0.001) compared with patients with a fair- or poor-signature microbiota.

## Predictive biomarkers of ICI combination therapy with VEGF inhibitors for HCC

Multiple lines of studies have validated the predictive value of many biomarkers in HCC patients receiving ICI combination therapy with VEGF inhibitors (Table 3). The studies conducted by Zhu et al. 42 and Kuzuya et al. 43 evaluated the on-treatment AFP change in blood of patients. The former study identified that high AFP decrease (≥ 75%) or low AFP increase (≤ 10%) predicted ORR (sensitivity/specificity, 59%/86%; 77%/44%, respectively) and longer median OS (NR vs. 14.2 months, P value < 0.001; 23.7 vs. 10.6 months, P value < 0.001, respectively) and PFS (13.2 vs. 6.7 months, P value < 0.001; 9.9 vs. 5.5 months, P value < 0.001, respectively) than low AFP decrease (< 75%) or high AFP increase (> 10%) in 150 HCC patients treated with PD-L1 ICI (atezolizumab) combined with VEGF inhibitor (bevacizumab); the latter study verified that low AFP ratio (< 1.4) predicted DCR (sensitivity/specificity, 89%/88%) and longer median PFS (30 vs. 6 weeks, P value = 0.0003) than high AFP ratio (≥ 1.4) in 50 HCC patients treated with PD-L1 ICI (atezolizumab) combined with VEGF inhibitor (bevacizumab). The study conducted by Chon et al. 44 measured the pre-treatment PIVKA-II level and NLR and on-treatment change in AFP and PIVKA-II levels and NLR in blood of 121 HCC patients treated with PD-L1 ICI (atezolizumab) combined with VEGF inhibitor (bevacizumab) and confirmed that high AFP decrease (≥ 30%) or PIVKA-II decrease (≥ 50%) or low NLR (< 2.5) predicted higher ORR (43% vs. 22%, P value < 0.05; 50% vs. 26%, P value < 0.05; 39% vs. 19%, P value < 0.05, respectively) than low AFP decrease (< 30%) or PIVKA-II decrease (< 50%) or high NLR (≥ 2.5); high NLR decrease (≥ 10%) predicted better OS than low NLR decrease (< 10%); low PIVKA-II level (< 186 mAU/mL) or NLR (< 2.5) predicted better OS and PFS than high PIVKA-II level (≥ 186 mAU/mL) or NLR (≥ 2.5). In addition, the study conducted by Campani et al. 45 combined the pre-treatment ALBI grade and on-treatment AFP change to stratify 70 HCC patients treated with PD-L1 ICI (atezolizumab) combined with VEGF inhibitor (bevacizumab) and showed that patients with low ALBI grade (grade 1) and high AFP decrease (≥ 20%) had the longest median OS (NR vs. 16.6 vs. 11.8 vs. 5.7 months, P value = 0.046) and PFS (NR vs. 8.6 vs. 5.6 vs. 2.3 months, P value = 0.012), followed by patients with high ALBI grade (grade 2) and high AFP decrease (≥ 20%), patients with low ALBI grade (grade 1) and low AFP decrease (< 20%), and patients with high ALBI grade (grade 2) and low AFP decrease (< 20%). The study conducted by Hatanaka et al. 46 combined the pre-treatment modified albumin-bilirubin (mALBI) grade and AFP level to stratify 426 HCC patients treated with PD-L1 ICI (atezolizumab) combined with VEGF inhibitor (bevacizumab) (255 patients as derivation cohort and 171 patients as validation cohort) and revealed that patients with low mALBI grade (1/2a) and low AFP level (< 100 ng/mL) had the highest OS rate (derivation cohort, 83% vs. 62% vs. 25%, P value < 0.001; validation cohort, 94% vs. 62% vs. 46%, P value < 0.001) and the longest median PFS (derivation cohort, 9.5 vs. 6.6 vs. 3.8 months, P value < 0.001; validation cohort, 9.3 vs. 6.7 vs. 4.7 months, P value = 0.018), followed by patients with high mALBI grade (2b/3) or high AFP level (≥ 100 ng/mL) and patients with high mALBI grade (2b/3) and high AFP level (≥ 100 ng/mL). Moreover, the study conducted by Yang et al. 47 detected the pre-treatment levels of several cytokines in blood of 165 HCC patients treated with PD-L1 ICI (atezolizumab) combined with VEGF inhibitor (bevacizumab) (84 patients as derivation cohort and 81 patients as validation cohort) and found that low interleukin-6 (IL-6) level (< 18.49 pg/mL) predicted higher ORR (derivation cohort, 38% vs. 0%; validation cohort, 29% vs. 7%) and longer OS (derivation cohort, P value = 0.021; validation cohort, P value < 0.001) and PFS (derivation cohort, P value = 0.003; validation cohort, P value = 0.018) than high IL-6 level (≥ 18.49 pg/mL). The study conducted by Giovannini et al. 48 analyzed the pre-treatment percentage of PD-1^+^ granulocytes in blood of 34 HCC patients treated with PD-L1 ICI (atezolizumab) combined with VEGF inhibitor (bevacizumab) and ascertained that low PD-1^+^ granulocyte percentage (< 13%) was associated with longer mean time to progression (TTP) (NR vs. 3.2 months, P value < 0.0001) than high PD-1^+^ granulocyte percentage (≥ 13%). The study conducted by Balcar et al. 49 examined the post-treatment immunoglobulin G (IgG) change in blood of 72 HCC patients treated with PD-1 ICI (such as nivolumab and pembrolizumab) or PD-L1 ICI (atezolizumab) combined with or without VEGF inhibitor (bevacizumab) and demonstrated that low IgG increase (< 14%) was correlated with longer median OS (15.9 vs. 6.4 months, P value = 0.001) and PFS (7.9 vs. 2.9 months, P value = 0.011) than high IgG increase (≥ 14%).

## Conclusions

This review comprehensively summarizes the evidence from the literature published so far which validate the predictive significance of a variety of biomarkers at different treatment time points (including pre-treatment, on-treatment, and post-treatment time points) in different sample sources (including tissue, blood, and stool samples) for the treatment responses and outcomes of HCC patients receiving different categories of ICI-based therapies (including ICI mono-therapy and combination therapy with other ICIs or TKIs or VEGF inhibitors) (Table 4). Among the current predictive biomarkers, most are derived from the blood and stool samples of HCC patients, supporting the convenience advantages of the use of noninvasive sampling methods in clinical application. Moreover, the clinical applicability varies among the predictive biomarkers. Certain biomarkers are selective for one category of ICI therapy at one specific treatment time point, such as tumoral PD-L1 expression level, intratumoral CD38^+^ cell proportion, ctDNA TMB or MSAF, PD-L1^+^ CTC count, PD-1^+^ granulocyte percentage, SII, PNI, and IgG change; in contrast, some biomarkers show predictive value for 2 or 3 different categories of therapies at 2 or 3 different treatment time points, such as NLR, PLR, AFP change, and PIVKA-II change. It should be carefully noted that even the same biomarker may have different cut-off values when applied for predicting HCC patients receiving different categories of therapies at different treatment time points. Besides, as a potential way to overcome the inter-patient heterogeneity among HCC patients, several biomarkers combine 2 different factors or even more factors in a nomogram to stratify the patients into more subgroups for prediction, such as CRP level and AFP level, PIVKA-II level and metastasis, ALBI grade and age, ALBI grade and AFP change, and mALBI grade and AFP level. In addition, many predictive biomarkers are based on immune cells or inflammatory cytokines, such as NLR, PLR, LMR, PD-1^+^ granulocyte percentage, SII, monocyte index, TGF-β, IL-6, and CRP, reflecting the clinical implication of tumor immune microenvironment in the efficacy of ICI therapy for HCC. Additionally, the predictive significance of the composition of gut microbiota, such as Erysipelotrichaceae, Veillonellaceae, Prevotella 9, and Lachnoclostridium, has been validated in HCC patients receiving ICI mono-therapy and combination therapy with TKIs. Considering the impact of gut microbiota-derived metabolites on ICI therapy for cancer 50-52, evaluation of the predictive significance of microbial metabolites in the blood and/or stool samples of HCC patients receiving ICI-based mono- and combination therapies may hold great promise to discover novel predictive biomarkers. Furthermore, non-coding RNAs such as microRNAs and long non-coding RNAs have been closely implicated in cancer and ICI therapy 53, 54. Whether non-coding RNAs can also serve as predictive biomarkers for ICI therapy in HCC patients is worth further investigation. Last but not the least, since the HCC patient cohorts evaluated in different studies may have different clinicopathological features and receive ICI-based therapies with different drugs (even though sharing the same molecular targets) at different treatment dosages, doses, and dosing intervals, it is quite important to take this issue into consideration when applying the predictive biomarkers to select the most suitable patient for the most suitable treatment for better therapeutic responses and outcomes.

## Figures and Tables

**Table 1:** Predictive biomarkers of ICI mono-therapy for HCC

Biomarkers	Source	Patients and Treatment	Predictive Significance^a^	Year	References
Pre-treatment tumoral PD-L1 expression level^b^	Tissue	195 HCC patients treated with PD-1 ICI	High PD-L1 expression level predicted longer OS.	2020	Sangro et al. 21
Pre-treatment TGF-β level^c^	Blood	28 HCC patients treated with PD-1 ICI	Low TGF-β level predicted longer OS and PFS.	2019	Feun et al. 22
Pre-treatment CRP and AFP levels^d^	Blood	101 HCC patients treated with PD-1 ICI	Patients with a low-risk score had the highest DCR and the longest OS and PFS, followed by patients with a medium- or high-risk score.	2022	Zhang et al. 23
Pre-treatment ALBI grade and age^e^	Blood	38 HCC patients treated with PD-1 ICI combined with locoregional therapy	Patients with a low-risk score had higher ORR and longer OS and PFS than patients with a high-risk score.	2022	Dong et al. 24
Pre-treatment NLR^f^	Blood	194 HCC patients treated with PD-1 ICI	Low NLR predicted longer OS and PFS.	2021	Choi et al. 25
Pre-treatment, on-treatment, or post-treatment NLR^g^	Blood	45 HCC patients treated with PD-1 ICI	Low pre-treatment, on-treatment, or post-treatment NLR predicted DCR and longer PFS.	2021	Hung et al. 26
Pre-treatment or post-treatment NLR or PLR^h^	Blood	103 HCC patients treated with PD-1 ICI combined with or without locoregional therapy	Low pre-treatment or post-treatment NLR predicted longer OS and PFS. Patients with a low pre-treatment or post-treatment PLR had the longest OS, followed by patients with a medium or high PLR.	2020	Dharmapuri et al. 27
Pre-treatment NLR, PLR, SII, or LMR^i^	Blood	110 HBV-related HCC patients treated with PD-1 ICI	Low NLR, PLR, or SII, or high LMR predicted longer OS and PFS.	2022	Huang et al. 28
On-treatment monocyte index^j^	Blood	45 HCC patients treated with PD-1 ICI	High monocyte index predicted DCR and longer PFS.	2023	Jeon et al. 29
Pre-treatment gut microbiota	Stool	30 HCC patients treated with PD-1 ICI	High abundance of Erysipelotrichaceae bacterium-GAM79 or low abundance of Veillonellaceae predicted longer OS.	2021	Mao et al. 30

^a^OS was defined as the time from treatment initiation to death due to any cause. PFS was defined as the time from treatment initiation to radiological progression or death due to any cause. ORR was defined as the proportion of patients with CR or PR. DCR was defined as the proportion of patients with CR, PR, or SD. ^b^Tumoral PD-L1 expression was defined as the percentage of PD-L1-expressing tumor cells in tumor tissues and was categorized as high (≥ 1%) or low (< 1%) level. ^c^TGF-β level was categorized as high (≥ 200 pg/mL) or low (< 200 pg/mL) level. ^d^The combination of CRP and AFP levels was categorized as high (CRP > 20.9 mg/L and AFP > 400 ng/mL), medium (CRP > 20.9 mg/L or AFP > 400 ng/mL), or low (CRP ≤ 20.9 mg/L and AFP ≤ 400 ng/mL) risk. ^e^ALBI grade was defined as log10 blood bilirubin level multiplied by 0.66 plus blood albumin level multiplied by -0.085 and was stratified as grade 1 (≤ -2.60), 2 (> -2.60 to ≤ -1.39), or 3 (> -1.39). The combination of ALBI grade and age was categorized as high (ALBI grade 2 and age < 53 years) or low (ALBI grade 1 and age ≥ 53 years) risk. ^f^NLR was calculated by dividing blood neutrophil count by blood lymphocyte count and was categorized as high (≥ 3) or low (< 3) ratio. ^g^On-treatment NLR was measured at day 14 after treatment initiation. Pre-treatment, on-treatment, and post-treatment NLR were categorized as high (> 2.5, ≤ 4.1, and > 2.7) or low (≤ 2.5, < 4.1, and ≤ 2.7) ratio, respectively. ^h^NLR was categorized as high (≥ 5) or low (< 5) ratio for pre-treatment and post-treatment. PLR was calculated by dividing blood platelet count by blood lymphocyte count and was divided into three level groups: low (≤ 118), medium (> 118 to < 224), and high (≥ 224) for pre-treatment; low (≤ 125), medium (> 125 to < 229), and high (≥ 229) for post-treatment. ^i^NLR was categorized as high (≥ 5) or low (< 5) ratio. PLR was categorized as high (≥ 140) or low (< 140) ratio. SII was calculated by multiplying blood platelet count by blood neutrophil count and dividing by blood lymphocyte count and was categorized as high (≥ 970) or low (< 970) index. LMR was calculated by dividing blood lymphocyte count by blood monocyte count and was categorized as high (≥ 1.8) or low (< 1.8) ratio. ^j^Monocyte index was calculated by dividing Monocyte_D7/D0_ by Monocyte-PDL1_D7/D0_, in which Monocyte_D7/D0_ was defined as the fold change in the frequency of classical monocytes at day 7 over day 0 after treatment initiation and Monocyte-PDL1_D7/D0_ was defined as the fold change in the frequency of PD-L1-expressing classical monocytes at day 7 over day 0 after treatment initiation in blood, and was categorized as high (≥ 1) or low (< 1) index. Abbreviations: ICI, immune checkpoint inhibitor; HCC, hepatocellular carcinoma; PD-L1, programmed death ligand 1; PD-1, programmed death 1; OS, overall survival; TGF-β, transforming growth factor-beta; PFS, progression-free survival; CRP, C-reactive protein; AFP, alpha-fetoprotein; DCR, disease control rate; ALBI, albumin-bilirubin; ORR, objective response rate; NLR, neutrophil to lymphocyte ratio; PLR, platelet to lymphocyte ratio; SII, systemic immune-inflammation index; LMR, lymphocyte to monocyte ratio; HBV, hepatitis B virus; CR, complete response; PR, partial response; SD, stable disease.

**Table 2:** Predictive biomarkers of ICI combination therapy with other ICIs or TKIs for HCC

Biomarkers	Source	Patients and Treatment	Predictive Significance^a^	Year	References
Pre-treatment intratumoral CD38^+^ cell proportion^b^	Tissue	49 HCC patients treated with PD-1 or PD-L1 ICI combined with or without CTLA-4 ICI	High CD38^+^ cell proportion predicted higher ORR and longer OS and PFS.	2020	Ng et al. 31
Pre-treatment PNI, NLR, or PLR^c^	Blood	362 HCC patients treated with PD-1 or PD-L1 ICI combined with or without CTLA-4 ICI	High PNI predicted higher DCR and longer OS. Low NLR predicted higher ORR and longer OS and PFS. Low PLR predicted longer OS and PFS.	2021	Muhammed et al. 32
On-treatment AFP change^d^	Blood	43 HCC patients treated with PD-1 ICI combined with or without CTLA-4 ICI	High AFP decrease predicted higher ORR and DCR and longer OS and PFS.	2019	Shao et al. 33
On-treatment AFP change^e^	Blood	75 HCC patients treated with PD-1 ICI combined with or without TKI	High AFP decrease predicted higher ORR and DCR and longer OS.	2020	Lee et al. 34
On-treatment AFP change^f^	Blood	95 HCC patients treated with PD-1 ICI combined with or without locoregional therapy or TKI	High AFP decrease predicted higher ORR and DCR and longer OS and PFS.	2021	Hsu et al. 35
Post-treatment AFP or PIVKA-II change^g^	Blood	235 HCC patients treated with PD-1 ICI combined with or without locoregional therapy and/or TKI	High AFP or PIVKA-II decrease predicted higher ORR and longer OS and PFS.	2021	Sun et al. 36
Pre-treatment nomogram based on ECOG PS, TACE, EHM, Child-Pugh score, ALT, AFP, and PLR^h^	Blood	258 HCC patients treated with PD-1 ICI combined with or without TKI	Patients with a low-risk score had the longest OS, followed by patients with a medium- or high-risk score.	2022	Li et al. 37
Pre-treatment PIVKA-II level and metastasis^i^	Blood	191 HCC patients treated with PD-1 ICI combined with TKI	Patients with a low-risk score had the longest OS, followed by patients with a medium- or high-risk score.	2023	Guo et al. 38
Pre-treatment ctDNA TMB or MSAF^j^	Blood	107 HCC patients treated with PD-1 ICI combined with TKI	Low ctDNA TMB predicted higher DCR and longer OS. Low ctDNA MSAF predicted higher DCR and longer OS.	2022	Xu et al. 39
Pre-treatment PD-L1^+^ CTC count^k^	Blood	47 HCC patients treated with PD-1 ICI combined with locoregional therapy and TKI	Low PD-L1^+^ CTC count predicted ORR and higher ORR and longer OS.	2022	Su et al. 40
Pre-treatment gut microbiota^l^	Stool	74 HCC patients treated with PD-1 ICI combined with or without TKI	Patients with a good signature of microbiota had the highest ORR and DCR and the longest OS and PFS, followed by patients with a fair or poor signature.	2022	Lee et al. 41

^a^OS was defined as the time from treatment initiation to death due to any cause. PFS was defined as the time from treatment initiation to radiological progression or death due to any cause. ORR was defined as the proportion of patients with CR or PR. DCR was defined as the proportion of patients with CR, PR, or SD. ^b^Intratumoral CD38^+^ cell proportion was defined as the percentage of CD38-expressing cells in tumor tissues and was categorized as high (≥ 5%) or low (< 5%) proportion. ^c^PNI was defined as blood albumin level plus 5 multiplies by blood lymphocyte count and was categorized as high (≥ 45) or low (< 45) index. NLR was calculated by dividing blood neutrophil count by blood lymphocyte count and was categorized as high (≥ 5) or low (< 5) ratio. PLR was calculated by dividing blood platelet count by blood lymphocyte count and was categorized as high (≥ 300) or low (< 300) ratio. ^d^AFP decrease was defined as the percentage of decrease in serum AFP levels at 4 weeks after treatment initiation relative to pre-treatment levels and was categorized as high (> 20%) or low (≤ 20%) decrease. ^e^AFP decrease was defined as the percentage of decrease in serum AFP levels at 4 weeks after treatment initiation relative to pre-treatment levels and was categorized as high (> 10%) or low (≤ 10%) decrease. ^f^AFP decrease was defined as the percentage of decrease in serum AFP levels at 3 months after treatment initiation relative to pre-treatment levels and was categorized as high (> 15%) or low (≤ 15%) decrease. ^g^AFP or PIVKA-II decrease was defined as the percentage of decrease in serum AFP or PIVKA-II levels after completion of treatment relative to pre-treatment levels and was categorized as high (> 50%) or low (≤ 50%) decrease. ^h^A total score was calculated based on the nomogram assigned ratio and was divided into three risk groups: low (≤ 182.7), medium (> 182.7 to ≤ 240.3), and high (> 240.3). ^i^The combination of PIVKA-II level and metastasis was categorized as high (PIVKA-II > 600 mAU/mL and with metastasis), medium (PIVKA-II > 600 mAU/mL or with metastasis), or low (PIVKA-II < 600 mAU/mL and without metastasis) risk. ^j^TMB was defined as the number of somatic mutations per megabase of sequenced ctDNA and was categorized as high (> 4) or low (≤ 4) burden. MSAF is an indicator of the amount of ctDNA in blood and was categorized as high (> 0.027) or low (≤ 0.027) frequency. ^k^PD-L1^+^ CTC count was categorized as high (≥ 2) or low (< 2) count. ^l^Good signature was defined as the coexistence of Prevotella 9 depletion and Lachnoclostridium enrichment, poor signature was defined as the coexistence of Prevotella 9 enrichment and Lachnoclostridium depletion, and fair signature was defined as the coexistence of both two bacteria depletion or both two bacteria enrichment. Abbreviations: ICI, immune checkpoint inhibitor; TKI, tyrosine kinase inhibitor; HCC, hepatocellular carcinoma; PD-1, programmed death 1; PD-L1, programmed death ligand 1; CTLA-4, cytotoxic T-lymphocyte associated antigen 4; ORR, objective response rate; OS, overall survival; PFS, progression-free survival; PNI, prognostic nutritional index; NLR, neutrophil to lymphocyte ratio; PLR, platelet to lymphocyte ratio; DCR, disease control rate; AFP, alpha-fetoprotein; PIVKA-II, protein induced by vitamin K absence or antagonist-II; ECOG PS, Eastern Cooperative Oncology Group performance status; TACE, transarterial chemoembolization; EHM, extrahepatic metastasis; ALT, alanine aminotransferase; ctDNA, circulating tumor DNA; TMB, tumor mutation burden; MSAF, maximum somatic allele frequency; CTC, circulating tumor cell; CR, complete response; PR, partial response.

**Table 3:** Predictive biomarkers of ICI combination therapy with VEGF inhibitors for HCC

Biomarkers	Source	Patients and Treatment	Predictive Significance^a^	Year	References
On-treatment AFP change^b^	Blood	150 HCC patients treated with PD-L1 ICI combined with VEGF inhibitor	High AFP decrease or low AFP increase predicted ORR and longer OS and PFS.	2022	Zhu et al. 42
On-treatment AFP change^c^	Blood	50 HCC patients treated with PD-L1 ICI combined with VEGF inhibitor	Low AFP ratio predicted DCR and longer PFS.	2022	Kuzuya et al. 43
Pre-treatment PIVKA-II level or NLR or on-treatment AFP, PIVKA-II, or NLR change^d^	Blood	121 HCC patients treated with PD-L1 ICI combined with VEGF inhibitor	High AFP or PIVKA-II decrease or low NLR predicted higher ORR. High NLR decrease predicted longer OS. Low PIVKA-II level or NLR predicted longer OS and PFS.	2023	Chon et al. 44
Pre-treatment ALBI grade and on-treatment AFP change^e^	Blood	70 HCC patients treated with PD-L1 ICI combined with VEGF inhibitor	Patients with low ALBI grade and high AFP decrease had the longest OS and PFS, followed by patients with high ALBI grade and AFP decrease, patients with low ALBI grade and AFP decrease, and patients with high ALBI grade and low AFP decrease.	2023	Campani et al. 45
Pre-treatment mALBI grade and AFP level^f^	Blood	426 HCC patients treated with PD-L1 ICI combined with VEGF inhibitor	Patients with low mALBI grade and AFP level had the highest OS rate and the longest PFS, followed by patients with high mALBI grade or AFP level and patients with high mALBI grade and AFP level.	2023	Hatanaka et al. 46
Pre-treatment IL-6 level^g^	Blood	165 HCC patients treated with PD-L1 ICI combined with VEGF inhibitor	Low IL-6 level predicted higher ORR and longer OS and PFS.	2023	Yang et al. 47
Pre-treatment PD-1^+^ granulocyte percentage^h^	Blood	34 HCC patients treated with PD-L1 ICI combined with VEGF inhibitor	Low PD-1^+^ granulocyte percentage predicted longer TTP.	2023	Giovannini et al. 48
Post-treatment IgG change^i^	Blood	72 HCC patients treated with PD-1 or PD-L1 ICI combined with or without VEGF inhibitor	Low IgG increase predicted longer OS and PFS.	2023	Balcar et al. 49

^a^OS was defined as the time from treatment initiation to death due to any cause. PFS was defined as the time from treatment initiation to radiological progression or death due to any cause. TTP was defined as the time from treatment initiation to radiological progression (but not death) due to any cause. ORR was defined as the proportion of patients with CR or PR. DCR was defined as the proportion of patients with CR, PR, or SD. ^b^AFP decrease or increase was defined as the percentage of decrease or increase in serum AFP levels at 4 weeks after treatment initiation relative to pre-treatment levels and was categorized as high (≥ 75%) or low (< 75%) decrease or high (> 10%) or low (≤ 10%) increase. ^c^AFP ratio was calculated by dividing serum AFP levels at 6 weeks after treatment initiation by pre-treatment levels and was categorized as high (≥ 1.4) or low (< 1.4) ratio. ^d^PIVKA-II level was categorized as high (≥ 186 mAU/mL) or low (< 186 mAU/mL) level. NLR was calculated by dividing blood neutrophil count by blood lymphocyte count and was categorized as high (≥ 2.5) or low (< 2.5) ratio. AFP, PIVKA-II, or NLR decrease was defined as the percentage of decrease in serum AFP, PIVKA-II, or NLR levels at the first response evaluation after treatment initiation relative to pre-treatment levels and was categorized as high (≥ 30%, ≥ 50%, or ≥ 10) or low (< 30%, < 50%, or < 10) decrease, respectively. ^e^ALBI grade was defined as log10 blood bilirubin level multiplied by 0.66 plus blood albumin level multiplied by -0.085 and was stratified as grade 1 (≤ -2.60), 2 (> -2.60 to ≤ -1.39), or 3 (> -1.39). ALBI grade 2 and 1 were defined as high and low ALBI grade, respectively. AFP decrease was defined as the percentage of decrease in serum AFP levels at 3 weeks after treatment initiation relative to pre-treatment levels and was categorized as high (≥ 20%) or low (< 20%) decrease. ^f^mALBI grade was calculated by the same formula as ALBI grade and stratified as grade 1 (≤ -2.60), grade 2a (> -2.60 to ≤ -2.27), grade 2b (> -2.27 to ≤ -1.39), or grade 3 (> -1.39). mALBI grade 2b/3 and 1/2a were defined as high and low mALBI grade, respectively. AFP level was categorized as high (≥ 100 ng/mL) or low (< 100 ng/mL) level. ^g^IL-6 level was categorized as high (≥ 18.49 pg/mL) or low (< 18.49 pg/mL) level. ^h^PD-1^+^ granulocyte percentage was defined as the percentage of PD-1-expressing granulocytes on total granulocytes in blood and was categorized as high (≥ 13%) or low (< 13%) percentage. ^i^IgG increase was defined as the percentage of increase in serum IgG levels at 6 weeks after treatment initiation relative to pre-treatment levels and was categorized as high (≥ 14%) or low (< 14%) decrease. Abbreviations: ICI, immune checkpoint inhibitor; VEGF, vascular endothelial growth factor; HCC, hepatocellular carcinoma; AFP, alpha-fetoprotein; PD-L1, programmed death ligand 1; ORR, objective response rate; OS, overall survival; PFS, progression-free survival; DCR, disease control rate; PIVKA-II, protein induced by vitamin K absence or antagonist-II; NLR, neutrophil to lymphocyte ratio; ALBI, albumin-bilirubin; mALBI, modified albumin-bilirubin; IL-6, interleukin-6; PD-1, programmed death 1; TTP, time to progression; IgG, immunoglobulin G; CR, complete response; PR, partial response.

**Table 4:** Comparative summary of predictive biomarkers of ICI-based mono- and combination therapy for HCC

Mono- and Combination Therapy		ICIs	ICIs and Other ICIs	ICIs and TKIs	ICIs and VEGF Inhibitors
ICI Targets		PD-1	PD-1 or PD-L1 and CTLA-4	PD-1	PD-L1
Pre-Treatment Biomarkers	Tissue	Tumoral PD-L1 expression level ↑^a^	Intratumoral CD38^+^ cell proportion ↑		
	Blood	NLR ↓^a^ PLR ↓ LMR SII ↓ TGF-β level ↓ CRP level ↓ and AFP level ↓ ALBI grade ↓ and age ↑	NLR ↓ PLR ↓ PNI ↑	ctDNA TMB ↓ ctDNA MSAF ↓ PD-L1^+^ CTC count ↓ PIVKA-II level ↓ and metastasis ↓ Nomogram based on ECOG PS, TACE, EHM, Child-Pugh score, ALT, AFP, and PLR ↓	NLR ↓ IL-6 level ↓ PIVKA-II level ↓ PD-1^+^ granulocyte percentage ↓ ALBI grade ↓ and AFP decrease ↑^b^ mALBI grade ↓ and AFP level ↓
	Stool	Gut microbiota Erysipelotrichaceae ↑ Veillonellaceae ↓		Gut microbiota Prevotella 9 ↓ Lachnoclostridium ↑	
On-Treatment Biomarkers	Blood	NLR ↓ Monocyte index ↑	AFP decrease ↑	AFP decrease ↑	AFP decrease ↑ AFP increase ↓ AFP ratio ↓ NLR decrease ↑ PIVKA-II decrease ↑
Post-Treatment Biomarkers	Blood	NLR ↓ PLR ↓		AFP decrease ↑ PIVKA-II decrease ↑	IgG increase ↓
Predictive Significance	Treatment responses	Higher ORR Higher DCR	Higher ORR Higher DCR	Higher ORR Higher DCR	Higher ORR Higher DCR
	Treatment outcomes	Longer OS Longer PFS	Longer OS Longer PFS	Longer OS Longer PFS	Longer OS Longer PFS Longer TTP

^a^Upward and downward arrows indicated that high and low levels of biomarkers predicted better treatment responses and outcomes, respectively. ^b^Although classified as a pre-treatment biomarker, this biomarker was based on pre-treatment ALBI grade and on-treatment AFP decrease in prediction. Abbreviations: ICI, immune checkpoint inhibitor; HCC, hepatocellular carcinoma; TKI, tyrosine kinase inhibitor; VEGF, vascular endothelial growth factor; PD-1, programmed death 1; PD-L1, programmed death ligand 1 NLR, neutrophil to lymphocyte ratio; PLR, platelet to lymphocyte ratio; LMR, lymphocyte to monocyte ratio; SII, systemic immune-inflammation index; TGF-β, transforming growth factor-beta; CRP, C-reactive protein; AFP, alpha-fetoprotein; ALBI, albumin-bilirubin; PNI, prognostic nutritional index; ctDNA, circulating tumor DNA; TMB, tumor mutation burden; MSAF, maximum somatic allele frequency; CTC, circulating tumor cell; PIVKA-II, protein induced by vitamin K absence or antagonist-II; ECOG PS, Eastern Cooperative Oncology Group performance status; TACE, transarterial chemoembolization; EHM, extrahepatic metastasis; ALT, alanine aminotransferase; IL-6, interleukin-6; mALBI, modified albumin-bilirubin; IgG, immunoglobulin G; ORR, objective response rate; DCR, disease control rate; OS, overall survival; PFS, progression-free survival; TTP, time to progression.
